# *Bacillus amyloliquefaciens SC06* Induced AKT–FOXO Signaling Pathway-Mediated Autophagy to Alleviate Oxidative Stress in IPEC-J2 Cells

**DOI:** 10.3390/antiox10101545

**Published:** 2021-09-28

**Authors:** Li Tang, Zihan Zeng, Yuanhao Zhou, Baikui Wang, Peng Zou, Qi Wang, Jiafu Ying, Fei Wang, Xiang Li, Shujie Xu, Pengwei Zhao, Weifen Li

**Affiliations:** 1 Key Laboratory of Molecular Animal Nutrition of the Ministry of Education, Institute of Feed Science, College of Animal Sciences, Zhejiang University, Hangzhou 310058, China; litang2017@zju.edu.cn (L.T.); 21817016@zju.ecu.cn (Z.Z.); 12017005@zju.edu.cn (Y.Z.); wangbaikui@zju.edu.cn (B.W.); 21917085@zju.edu.cn (P.Z.); 21917012@zju.edu.cn (Q.W.); 21817401@zju.edu.cn (J.Y.); 22017007@zju.edu.cn (F.W.); 22017014@zju.edu.cn (X.L.); 22017075@zju.edu.cn (S.X.); 2 Key Laboratory of Animal Nutrition and Feed Science (Eastern of China), Ministry of Agriculture and Rural Affairs, Hangzhou 310058, China; 3 Department of Biochemistry, Department of Cardiology of Second Affiliated Hospital, Zhejiang University School of Medicine, Hangzhou 310058, China

**Keywords:** *Bacillus amyloliquefaciens SC06*, IPEC-J2, oxidative stress, autophagy, apoptosis, AKT–FOXO

## Abstract

Autophagy is a conserved proteolytic mechanism, which degrades and recycles damaged organs and proteins in cells to resist external stress. Probiotics could induce autophagy; however, its underlying molecular mechanisms remain elusive. Our previous study has found that *BaSC06* could alleviate oxidative stress by inducing autophagy in rats. This research aimed to verify whether *Bacillus amyloliquefaciens SC06* can induce autophagy to alleviate oxidative stress in IPEC-J2 cells, as well as explore its mechanisms. IPEC-J2 cells were first pretreated with 10^8^ CFU/mL *BaSC06*, and then were induced to oxidative stress by the optimal dose of diquat. The results showed that *BaSC06* significantly triggered autophagy, indicated by the up-regulation of LC3 and Beclin1 along with downregulation of p62 in IPEC-J2 cells. Further analysis revealed that *BaSC06* inhibited the AKT–FOXO signaling pathway by inhibiting the expression of p-AKT and p-FOXO and inducing the expression of SIRT1, resulting in increasing the transcriptional activity of FOXO3 and gene expression of the ATG5–ATG12 complex to induce autophagy, which alleviated oxidative stress and apoptosis. Taken together, *BaSC06* can induce AKT–FOXO-mediated autophagy to alleviate oxidative stress-induced apoptosis and cell damage, thus providing novel theoretical support for probiotics in the prevention and treatment of oxidative damage.

## 1. Introduction

The gastrointestinal tract is an important primary digestive organ, whilst intestinal health directly affects the health and growth of animals. While exposed to exogenous stimulators, the gastrointestinal tract is susceptible to oxidative stress [1,2]. The mucosal barrier, composed of intestinal epithelium, the mucus layer, and cells involved in local immune responses [3], plays a key role in maintaining intestinal homeostasis. The epithelium is composed of a single layer of columnar intraepithelial cells. IPEC-J2 cells, derived from the columnar epithelial cells of a piglet’s jejunum, were first isolated from the middle jejunum of neonatal piglets by A.J. Brosnahan et al. at the University of North Carolina [4]. IPEC-J2 cells were initially used as a porcine small intestine model to study the pathogenic bacteria that induce porcine hyperplastic bowel disease [5], and then gradually applied to various studies, including oxidative stress [6], intestinal microorganism [7], and intestinal immune response [8]. IPEC-J2 cells are highly similar to normal intestinal epithelial cells due to their tumor-free genes, and are derived from piglets. The high similarity between IPEC-J2 cells and the real pig and human small intestine has gradually attracted researchers’ attention in recent years.

Autophagy is an evolutionary conserved cell procedure that participates in the lysosomal degradation of proteins and organelles, contributing significantly to cell growth maintenance, differentiation, and homeostasis in some adverse conditions, such as hypoxia and starvation [9]. It plays an integral role in maintaining cellular homeostasis and promoting cell survival, while apoptosis exerts a defensive role, selectively removing cells and renewing cells and tissues [10,11]. Therefore, an equilibrium of autophagy and apoptosis in intestinal epithelial cells affects intestinal health. During the process of autophagy, portions of the cytoplasm or the entire organelle are sequestered in double-membranous vesicles, called autophagy vacuoles (AV) or autophagosomes. The autophagosome then fuses with the lysosome to form a monofilm autophagosome that degrades its contents [12].

In vivo and in vitro models, increasing evidence reveals that probiotics have the potential to alleviate oxidative stress in livestock and prevent them from oxidative stress. Some probiotics, such as *Lactobacillus and Bifidobacterium*, exhibit a good therapeutic effect on alleviating intestinal oxidative stress [13,14,15]. Probiotic *Bacillus* spp. are widely used to improve animal growth performance and prevent gastrointestinal disorders [16,17] and significantly alleviated intestinal oxidative stress in aquatic products and piglets [18,19,20]. In addition, *Bacillus* spp. could prevent oxidative stress and LPS-induced inflammatory responses in Raw 264.7 macrophages [21]. Furthermore, in chickens, *Bacillus subtilis* increased their antioxidant capacity and oxidative stability [22]. Studies have elucidated that probiotic can protect intestinal epithelial cells from damage and necrotizing apoptosis by regulating the autophagy and apoptosis signaling pathways, recruiting immune cells, and anti-inflammatory factors [23,24]. Some recent studies have shown that probiotics can regulate autophagy to alleviate oxidative stress. For example, metronidazole and *L. reuteri* combination treatment could decrease oxidative stress and inflammatory and autophagic pathways to prevent NAFLD progression [25]. *Lactobacillus reuteri* *ZJ617* and *Lactobacillus rhamnosus GG* supplementation suppressed lipopolysaccharide-induced oxidative stress by attenuating apoptosis and autophagy via the mTOR signaling pathway [26].

Currently, studies on the effect of probiotics on oxidative stress by regulating autophagy are still rare. In our previous experiments involving rats, it was uncovered that *Bacillus amyloliquefaciens SC06* (*BaSC06*) alleviated oxidative stress through autophagy via the p38 signaling pathway, but the other specific signaling pathway was inadequately investigated [27]. This study therefore sought to verify whether *BaSC06* can induce autophagy to alleviate oxidative stress in IPEC-J2 cells, as well as explore the other related signaling pathways.

## 2. Materials and Methods

### 2.1. BaSC06 Bacterial Strain Preparation

For this study, the probiotic *BaSC06* (CCTCC No: M2012280) was isolated from soil by the Laboratory of Microbiology, Institute of Feed Sciences, Zhejiang University, and preserved at the China Center for Type Culture Collection Afterward, the *BaSC06* strains were cultured at 37 °C in Luria-Bertani (LB) broth overnight, and then gathered by centrifugation (8000 rpm for 5 min). After that, the *BaSC06* strains were washed twice with PBS (pH = 7.4) and suspended at 10^8^ CFU/mL in the cell culture media. The fresh bacteria suspensions were prepared for cell incubation.

### 2.2. IPEC-J2 Cell Culture

IPEC-J2 cells were provided by Northwest Sci-Tech University of Agriculture and Forestry, which was then incubated at 37 °C in a humidified 5% CO_2_ with DMEM/F12 (HyClone, Logan, UT, USA) media, containing 10% FBS (Gibco, Grand Island, NE, USA) and 1% antibiotics (100 mg/mL of streptomycin and 100 U/mL of penicillin G).

### 2.3. Establishing Oxidative Stress Model in IPEC-J2 Cells

The diquat (DQ)-induced oxidative stress model was evaluated utilizing an MTT cell assay kit for cell proliferation and cytotoxicity (Nanjing Jiancheng Bioengineering Institute, Nanjing, China). According to instructions, 10^4^ cells per well were seeded in 96-well plates and cultured for 12 h, followed by DQ treatment at various concentrations (0, 250, 500, 750, 1000, and 1250 µmol/L) for 6 h, with nine parallel holes in each group. Thereafter, to each well was added 50 µL of MTT assay solution, and then incubated for 4 h. Afterward, a Spectra Max M5 microplate reader (Sunnyvale, CA, USA) was used to determine the absorbance of the plate at 570 nm. In order to set up the oxidative stress model for IPEC-J2 cells, the optimal DQ concentration was selected according to the IC_50_, calculated using a probability unit based on the MTT assay. The optimal concentration of *BaSC06* was determined using a Cell Counting Kit-8 (CCK-8 kit, Nanjing Jiancheng Bioengineering Institute, Nanjing, China) as per the instructions of the manufacturer. IPEC-J2 cells were treated with *BaSC06* at various concentrations (0, 10^5^, 10^6^, 10^7^, 10^8^, and 10^9^ CFU/mL) using the same seeding method as MTT, with nine parallel holes in each group, for 6 h. A total of 10 µL of CCK-8 solutions were added to every well, and the plate incubated for 1 h. The optimal *BaSC06* concentration was selected and calculated according to the viability of the cells based on the CCK-8 assay. The Spectra Max M5 microplate reader (Sunnyvale, CA, USA) was used to determine the absorbance of the plate at 450 nm.

IPEC-J2 cells were further divided into four groups: CK (PBS treatment only), DQ (diquat (DQ) treatment only), Ba (*BaSC06* treatment only), and Ba+DQ (*BaSC06* combined with diquat treatment) groups. Notably, IPEC-J2 cells in the CK group were treated with PBS, while those in the DQ and Ba groups were treated with 950 μmol/mL diquat and 10^8^ CFU/mL *BaSC06*, respectively. In the Ba+DQ group, IPEC-J2 cells was pretreated with 10^8^ CFU/mL *BaSC06* for 6 h and then treated with 950 μmol/mL diquat for the same period. Before *BaSC06* or DQ treatment, each group was washed twice with PBS at the same time.

Furthermore, 25 µM AKT phosphorylation inhibitor Perifosine (Biotime Biotechnology) was used to pre-incubate with IPEC-J2 cells for 48 h according to the instruction, and we then repeated the former *BaSC06* and DQ treatment methods.

### 2.4. ROS Generation Analysis

A reactive oxygen species (ROS) assay kit (Nanjing Jiancheng Bioengineering Institute, Nanjing, China) was utilized to detect the ROS level in treated IPEC-J2 cells in 96-well plates, 10^4^ cells in one well, with nine parallel holes in each group. The 2′,7′-dichlorohydro-fluorescein diacetate (DCFH-DA) is the most sensitive and commonly used probe for detecting intracellular ROS. Specifically, all cell samples were treated with 10 µM DCFH-DA solution, after pretreated with *BaSC06* and DQ at 37 °C for 30 min and subsequently washed with FBS-free media and PBS. DCFH is oxidized into a strong green fluorescence substance DCF (dichlorofluorescein) in the presence of ROS in cells, and its emission wavelength is 502 nm, while the fluorescence peaks at the wavelength of 530 nm. Its fluorescence intensity is proportional to the ROS levels in cells. The fluorescence signals were monitored using the microplate reader SpectraMax M5 (Sunnyvale, CA, USA) and an Olympus BX61W1-FV1000 laser scanning confocal microscope (Tokyo, Japan).

### 2.5. Detection of Antioxidant Capacities

IPEC-J2 cell lysates were gathered to determine oxidative stress indexes including the level of methane dicarboxylic aldehyde (MDA), and the activities of superoxide dismutase (SOD), catalase (CAT), as well as the glutathione peroxidase (GSH-Px), each group with 6 replications. All assays were performed following the manufacturer’s guidelines (Jiancheng Bioengineering Institute, Nanjing, China) [28].

### 2.6. Apoptosis Cell Analysis by TUNEL Assay

For this experiment, an Apoptosis Detection Kit named TUNEL BrightGreen (Vazyme, Nanjing, China) was used to detect the apoptosis levels in IPEC-J2 cells (*n* = 6). The 3′-hydroxyl terminus of the broken DNA can bind to FITC-12-DUTP, which is activated by Terminal Deoxynudleotidyl Transferase (TdT). Based on the manufacturer’s instruction, 4% paraformaldehyde was used to fix the slides. Then all the slides were incubated with proteinase K solution at a concentration of 20 µg/mL, followed by 50 µL BrightGreen labeling mix as well as 50 µL recombinant TDT enzyme. Then, the stained IPEC-J2 cells were instantly examined under an Olympus BX61W1-FV1000 laser scanning confocal microscope (Tokyo, Japan). Furthermore, IPEC-J2 cell were collected to be measured by a FC500 flow cytometer (Beckman Coulter, Fullerton, CA, USA).

### 2.7. Annexin V-FITC/PI Apoptosis Assay

Annexin V has been used as a sensitive indicator of early apoptosis because it binds to the membrane of early apoptotic cells via phosphatidylserine exposed externally. Propidium iodide (PI) is a nucleic acid dye that cannot penetrate the entire cell membrane, but PI can penetrate the cell membrane and make the nucleus red due to increased membrane permeability in the middle and late apoptotic cells as well as dead cells. Therefore, annexin V matched with PI could be used to distinguish cells at different stages of apoptosis. IPEC-J2 cells were collected and washed in PBS. Then they were suspended in a 1× annexin binding buffer, mixed, and incubated with 5 μL annexin V-FITC V and 5 μL PI for 10 min. After that, a FC500 flow cytometer (Beckman Coulter, Fullerton, CA, USA) was utilized to measure the stained cells.

### 2.8. Detection of Caspase-3 Activity

The activity of caspase-3 was measured by the Caspase-3 Activity Kit (Beyotime, Shanghai, China). After treatment, IPEC-J2 cells were collected after rinsed with cold PBS and centrifugation. Caspase-3 activity in the supernatant were assayed using the kit following the instruction. The caspase activity was expressed as the percentage of enzyme activity compared to the control.

### 2.9. FOXO3a siRNA and Transfection

Three small interfering RNAs targeting pig FOXO3a and negative control siRNA were synthesized by Sangon Biotech, Shanghai, China, the sequences are summarized in Table 1. IPEC-J2 cells were cultured in antibiotic-free medium, then treated with Lipo-2000 and siRNA mixture. All the cells were collected for western blotting. 

### 2.10. Western Blotting

We refer to the protocol of Xiao et al. for our Western blot assay [29]. The cell lysis buffer produced by Western and American Psychological Association (RIPA, Biotime Biotechnology, Xiamen, China) was used to prepare the total IPEC-J2 cell lysates (*n* = 3), while the nuclear protein extraction kit (Biotime Biotechnology, Xiamen, China) was utilized to gather nuclear proteins (*n* = 3). In accordance with the manufacturer’s protocol, a BCA Protein assay kit (Biotime Biotechnology, Xiamen, China) was used to measure protein concentrations. After the SDS-PAGE, proteins were electrophoretically transferred to nitrocellulose membranes (Sangon Biotech, Shanghai, China). Subsequently, the membranes were incubated with first antibodies at 4 °C overnight, and thereafter incubated with secondary antibodies goat anti-mouse IgG-HRP and goat anti-rabbit IgG-HRP after washing by TBST. The blots were then developed with an ECL detection system (Tanon 5200, Shanghai, China). Anti-Bcl2 and anti-LC3II primary antibodies were purchased from Abcam (Cambridge, MA, USA), while primary antibodies such as anti-Bax, anti-Beclin1, anti-SQSTM/p62, anti-phosphor-mTOR (Ser2448), anti-mTOR, anti-phosphor-AKT (Ser473), and anti-AKT used in this study were obtained from CST (Danvers, MA, USA). Additionally, anti-FOXO3, anti-phosphor-FOXO3 (Ser253), and anti-Histone1 were acquired from Huabio (Hangzhou, China). Mouse anti-β-actin monoclonal antibody was obtained from Biotime Biotechnology, Xiamen, China. For all the proteins mentioned above, the relative density was analyzed with ImageJ software.

### 2.11. Immunofluorescence Analysis

IPEC–J2 cells were seeded and cultured in 12-well plates for 12 h to reach 70% confluence. Cell samples (*n* = 6) were fixed with cold methanol for 5 min, and after that, all the samples were blocked with 2.5% BSA at room temperature for 2 h, followed by incubating with anti-LC3II antibody (Abcam, Cambridge, MA, USA) for 12 h at 4 °C. Thereafter, Alexa Fluor 488 conjugated antibody (Biotime Biotechnology, Xiamen, China) and DAPI solution (Biotime Biotechnology, Xiamen, China) were used to stain cells and nucleus. An Olympus BX61W1-FV1000 laser scanning confocal microscope (Tokyo, Japan) was utilized to obtain images.

### 2.12. RNA Extractions and Quantitative Real-Time PCR (qPCR) Analysis

Total RNA was extracted utilizing RNAiso plus (Takara, Japan) from IPEC-J2 cells. The Nanodrop was used to examine the concentration of RNA. We utilized the PrimeScript II 1st Strand cDNA Synthesis Kit (Vazyme, Nanjing, China) to synthesize cDNA based on the manufacturer’s manual. The qRT-PCR was conducted with the use of the HiScript II One Step qRT-PCR SYBR Green Kit (Vazyme, Nanjing, China) based on the manufacturer’s manual. Glyceraldehyde-3-phosphate dehydrogenase (GAPDH) was used to normalize the amount of total RNA as an endogenous control. The Primer 5.0 as well as the Oligo 7.0 software were utilized to design and validate the primers. The primer sequences are summarized in Table 2. We estimated the abundance of the mRNA using the 2^−ΔΔCt^ method.

### 2.13. RNA Extraction and RNA-Seq Analysis

After the total RNA extraction, mRNA was purified from total RNA using poly-T oligo-attached magnetic beads. First-strand cDNA was synthesized using random primers. Second-strand cDNA was synthesized by DNA polymerase I, Rnase H, dNTP, and buffer. Then, the cDNA fragments were purified with an AMPure XP system (Beckman Coulter, Beverly, GA, USA). Afterwards, PCR was performed with Phusion High-Fidelity DNA polymerase, Universal PCR primers, and Index (X) Primer. Lastly, the PCR products were purified (AMPure XP system) and the library quality assessed on an Agilent Bioanalyzer 2100 system. The ligated products were size-selected by agarose gel electrophoresis and PCR, and then sequenced using Illumina HiSeqTM 4000. For the bioinformatics analysis, the original reading containing the adapter or low quality (Q value ≤ 10) was removed and mapped to the pig reference genome (assembly SSCROFA 11–1). We further analyzed the differentially expressed genes (DEGs) between different samples or groups with the DeSeq2 R package (1.16.1). Genes with a fold change of ≥1, at a false discovery rate of *p* ≤ 0.05, were considered significantly differentially expressed. The enrichment of DEGs was performed using the KEGG pathway database.

### 2.14. Statistical Analysis

All data are showed in the form of the mean ± standard deviation (SD). Statistically significant differences between means were calculated with a one-way ANOVA with a Tukey test and analyzed in SPSS statistical software, version 23.0 (IBM®, Chicago, IL, USA). The IC_50_ of the DQ to IPEC-J2 cells was calculated utilizing the probit method in SPSS statistical software, version 23.0 (Chicago, IL, USA). *p* < 0.05 was considered statistically significant. Figures were prepared utilizing Prism 9.0 software (GraphPad Software Inc., San Diego, CA, USA) and Origin 8.0 software (Origin Lab Corporation, Northampton, MA, USA).

## 3. Results

### 3.1. Establishment of Oxidative Stress Model Induced by Diquat in IPEC-J2 Cells

**Figure 1:**
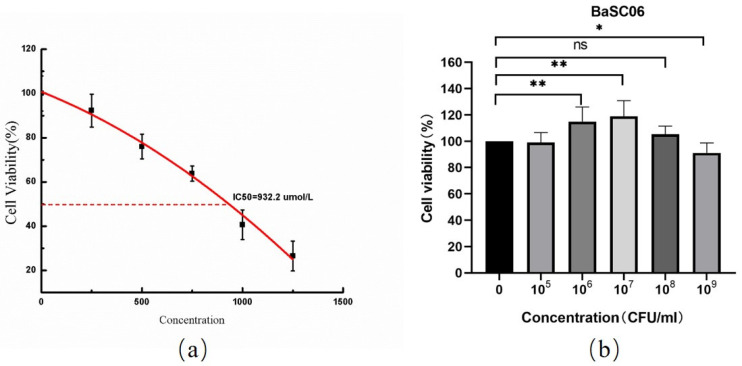
The establishment of a diquat-induced oxidative stress model. (**a**) IPEC-J2 cells were treated with DQ at various concentrations (0, 250, 500, 750, 1000, 1250 μmol/L) for 6 h. The IC_50_ was calculated using the probit method. (**b**) IPEC-J2 cells were treated with *BaSC06* for 6 h at different concentrations (0, 10^5^, 10^6^, 10^7^, 10^8^, and 10^9^ CFU/mL). Cell viability was calculated using an CCK-8 kit. Data are exhibited in the form of the mean ± SD (*n* = 9), and significance was measured by one-way ANOVA with a Tukey test: * *p* < 0.05, ** *p* < 0.01, and ns = no significance (*p* > 0.05).

IC_50_ represents 50% of the inhibitor concentration required for inhibition of enzymes, cells, cell receptors, or microorganisms. DQ, which is widely utilized as an herbicide in agriculture, was used to establish an oxidative stress model. Notably, DQ reduced the IPEC-J2 cells’ viability in a dose-dependent manner (Figure 1a). Utilizing the probit method, the IC_50_ for DQ in IPEC-J2 cells was 932.2 μmol/mL. Therefore, 950 μmol/mL DQ was used in the following experiment. IPEC-J2 cells were exposed to *BaSC06* at different concentrations (0, 10^5^, 10^6^, 10^7^, 10^8^, and 10^9^ CFU/mL) for 6 h, and their cell viability was further detected. As shown in Figure 1b, the cell viability at the 10^8^ CFU/mL treatment was the closest to 100% (105.384%), showing no significant change compared to the cells in the CK group (*p* > 0.05). Hence, 10^8^ CFU/mL *BaSC06* was used in further experiments.

### 3.2. BaSC06 Alleviated Oxidative Stress Induced by DQ in IPEC-J2 Cells

**Figure 2:**
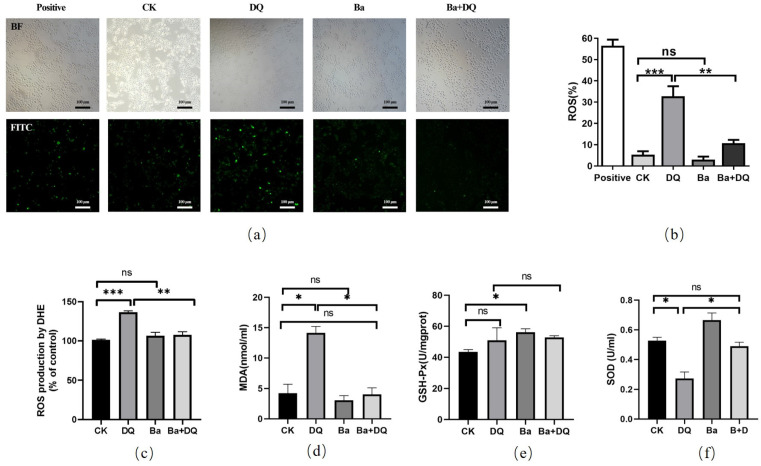
*BaSC06* alleviated oxidative stress induced by DQ in IPEC-J2 cells. (**a**–**c**) Levels of ROS measured by DCFH-DA fluorescence assay, and the data are presented as the ratio of green fluorescence and the percentage of ROS production by DHE. Scale bar: 100 μm; *n* = 9 in each group. (**d**–**f**) Antioxidant capacity in cell lysates indicated by MDA, T-SOD, and GSH-Px; *n* = 6 in each group. All data were analyzed utilizing one-way ANOVA with Tukey test: * *p* < 0.05, ** *p* < 0.01, *** *p* < 0.001, and ns = no significance (*p* > 0.05).

Consequent ROS generation and redox cycling are believed to be the key causes of oxidative stress induced by DQ [30]. The production of ROS in IPEC-J2 cells was examined using a DCFH-DA fluorescence assay and Microplate Reader. Compared with the CK group, DQ treatment significantly increased the generation of ROS (32.73 ± 4.755%, *p* < 0.001), which was markedly inhibited by *BaSC06* pretreatment (10.70 ± 1.588%, *p* < 0.01) (Figure 2a,b). A similar result detected by microplate reader was obtained (Figure 2c). In addition, MDA content was significantly increased in the DQ group (14.12 ± 1.07 nmol/mg protein, *p* < 0.01) (Figure 2d), but markedly decreased in the Ba+DQ group (4.03 ± 1.07 nmol/mg protein, *p* < 0.01). However, no significant difference was observed in the GSH-Px activity between CK and DQ groups, which was significantly increased in *BaSC06*-treated cells (*p* < 0.05) (Figure 2e). At the same time, compared with the CK group, the activity of T-SOD decreased by 48.2% (*p* <0.05) in the DQ group, which significantly increased by 26.3% in *BaSC06* pretreatment group (*p* < 0.05) (Figure 2f). These results suggest that *BaSC06* could enhance the antioxidant capacity of IPEC-J2 cells by decreasing the production of ROS and increasing the activities of antioxidant enzymes.

### 3.3. BaSC06 Alleviated Oxidative Stress-Induced Apoptosis in IPEC-J2 Cells

**Figure 3:**
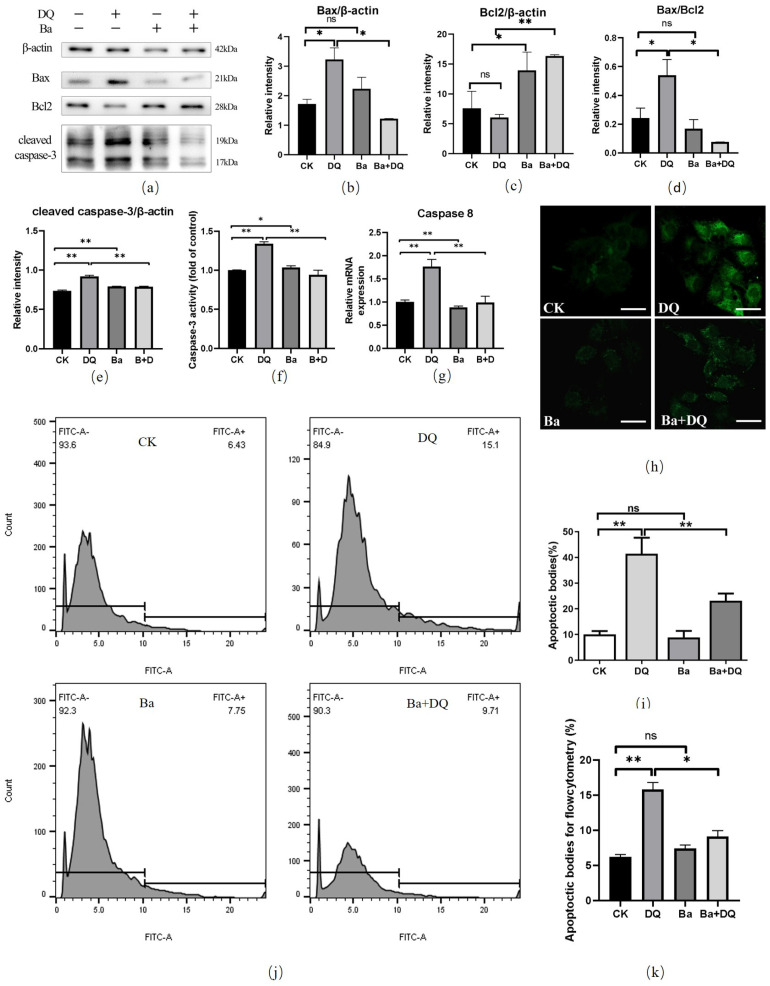

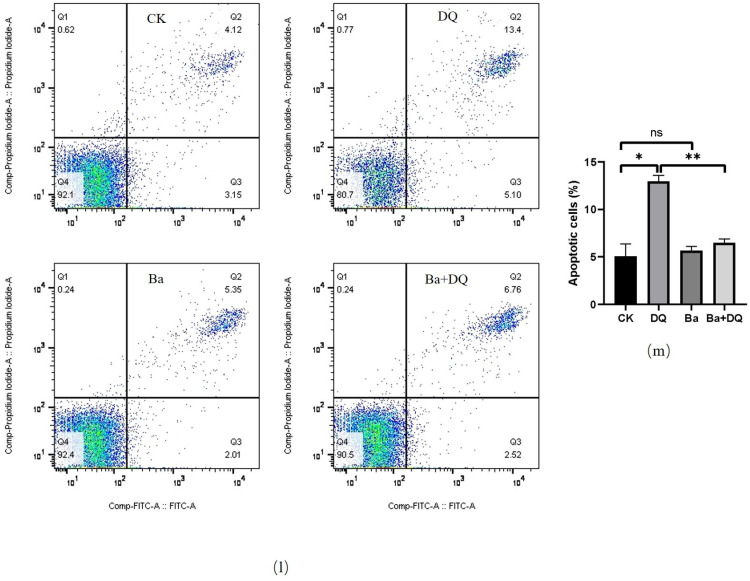
*BaSC06* pretreatment inhibited apoptosis in IPEC-J2 cells during diquat exposure. (**a**–**e**) The ratio of Bcl2/β-actin, Bax/β-actin and Bax/Bal2, and cleaved caspase-3/β-actin were analyzed using ImageJ software. Data were analyzed utilizing one-way ANOVA with a Tukey test; *n* = 3 in each group; * *p* < 0.05, ** *p* < 0.01, and ns = no significance (*p* > 0.05). (**f**) Caspase-3 activity was evaluated using a caspase-3 activity assay kit. (**g**) The mRNA expression levels of caspase-8 were determined using quantitative real-time PCR. (**h**,**i**) TUNEL assay. IPEC-J2 cells were stained with a BrightGreen apoptosis detection kit, after being treated with 10^8^ CFU/mL BaSC06 and 950 μmol/mL diquat. Scale bar: 5 μm. (**j**,**k**) TUNEL assay by flow cytometry. (**l**,**m**) Annexin V-FITC/PI apoptosis assay. Apoptotic cell rates were detected with a FITC annexin V-FITC/PI apoptosis kit, and then analyzed by flow cytometry. All data were analyzed using one-way ANOVA with a Tukey test; *n* = 6 in each group; * *p* < 0.05, ** *p* < 0.01, and ns = no significance (*p* > 0.05).

The results of the expression of apoptosis-related proteins showed that *BaSC06* markedly upregulated the protein level of Bcl2 (*p* < 0.05), while that in the DQ treatment group indicated no significant changes compared with untreated cells (*p* > 0.05) (Figure 3a,c). The expression of Bax and the ratio of Bax/Bcl2 was significantly increased in cells treated with DQ, but *BaSC06* pre-treatment altered this trend significantly (*p* < 0.05) (Figure 3a,b,d). DQ also increased the activity of caspases-3 (Figure 3f), the relative intensity of cleaved caspase-3 and the mRNA levels of caspase-8 considerably (*p* < 0.01), which were significantly reversed by BaSC06 pre-treatment (*p* < 0.01, Figure 3e,g). In addition, compared with the CK group, the number of apoptotic bodies (green puncta) in the DQ group, detected using a TUNEL kit, was considerably increased by 31.40 ± 7.19% (*p* < 0.01), but no significant changes and a significant decrease (18.25 ± 5.843%) were observed in the Ba group and Ba+DQ group (*p* < 0.01), respectively (Figure 3h,i). Furthermore, the similar results were obtained using TUNEL assay by flow cytometry (Figure 3j,k). *BaSC06* pretreatment dramatically downregulated DQ-triggered apoptosis (*p* < 0.01). The percentages of early and late apoptotic cells in the CK, DQ, Ba, and Ba+DQ groups were 5.05 ± 1.31%, 12.95 ± 0.64%, 5.67 ± 0.45%, and 6.48 ± 0.40%, respectively (Figure 3l,m). Overall, these findings demonstrate that pre-treatment with *BaSC06* might significantly alleviate apoptosis induced by DQ in IPEC-J2 cells.

### 3.4. BaSC06 Triggered Autophagy during Oxidative Stress in IPEC-J2 Cells

**Figure 4:**
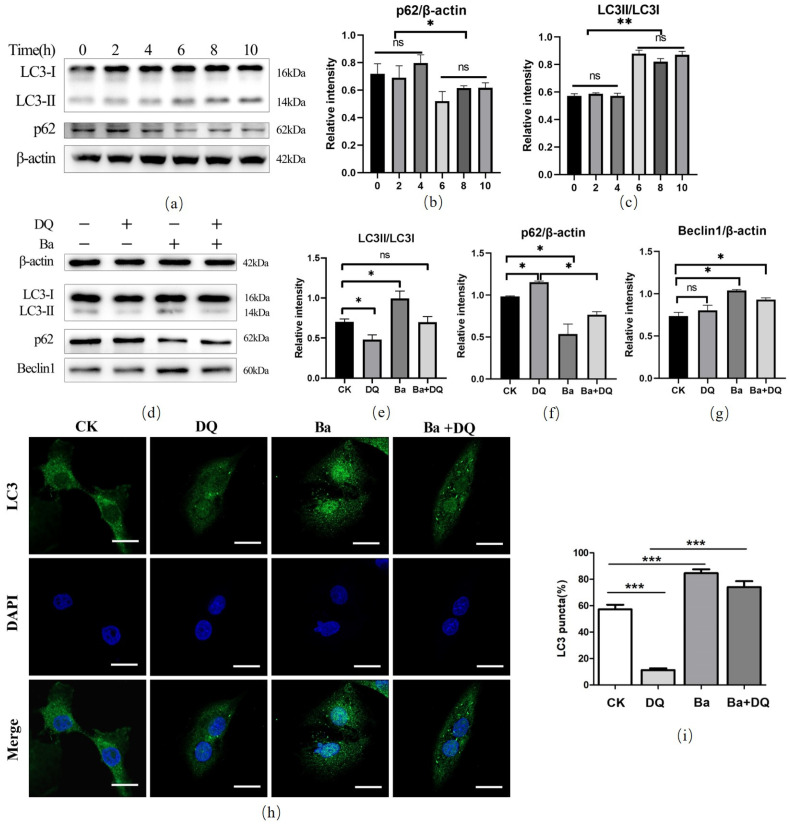
Effects of *BaSC06* on autophagy during oxidative stress in IPEC-J2 cells. (**a**–**c**) *BaSC06* triggered autophagy in IPECJ-J2 cells in a time-dependent manner. Cell lysates were collected to detect the protein levels of LC3-II/LC3-I and p62/β-actin, and data were analyzed using ImageJ software. (**d**–**g**) IPEC-J2 cells pretreated with *BaSC06* in the concentration of 10^8^ CFU/mL for 6 h, subsequently treated with DQ in the concentration of 950 μmol/mL for another 6 h. The LC3II/LC3I, p62/β-actin, or Beclin1/β-actin ratio was analyzed by ImageJ software. Data were analyzed using one-way ANOVA with a Tukey test; *n* = 3 in each group; * *p* < 0.05, ** *p* < 0.01, and ns = no significance (*p* > 0.05). (**h**,**i**) *BaSC06* increased LC3 puncta in IPEC-J2 cells. After the same treatment with (**d**), IPEC-J2 cells were stained and visualized under confocal microscopy for immunofluorescence analysis. Scale bar: 5 μm; *n* = 6 in each group. The number of LC3-positive cells was statistically analyzed: * *p* < 0.05, ** *p* < 0.01, *** *p* < 0.001, and ns = no significance (*p* > 0.05).

We further evaluated whether *BaSC06* can induce autophagy in IPEC-J2 cells under oxidative stress. As expected, 10^8^ CFU/mL *BaSC06* upregulated the LC3II/LC3I ratio dramatically in a time-dependent manner, particularly from 6 h to 10 h (*p* < 0.05) (Figure 4a,b), but decreased p62 expression (Figure 4a,c). A significant decreased expression of LC3-II in the DQ and Ba+DQ group was found (*p* < 0.05) (Figure 4d,e), and no significant change was observed between the two groups (*p* > 0.05). Further, DQ treatment substantially inhibited the degradation of p62 (*p* < 0.05); however, *BaSC06* pretreatment dramatically blocked this trend (*p* < 0.05) (Figure 4d,f). The ratio of LC3II/LC3I markedly increased in the *BaSC06* group compared to the DQ group (*p* < 0.01) (Figure 4d,e). Additionally, compared with the CK group, *BaSC06* pretreatment markedly upregulated the expression of Beclin1 (*p* < 0.05), while that in the DQ group exhibited no significant change (*p* > 0.05) (Figure 4d,g). The results of autophagy based on the percentage of LC3-positive cells demonstrated that, compared with the CK group, DQ significantly downregulated LC3-positive cells (45.92 ± 3.23%, *p* < 0.01), while *BaSC06* significantly increased LC3 puncta (62.81 ± 4.90%, *p* < 0.001) (Figure 4h,i). To sum up, these findings suggest that *BaSC06* treatment can induce autophagy in IPEC-J2 cells.

### 3.5. KEGG Pathway Analysis of DEGs

**Figure 5:**
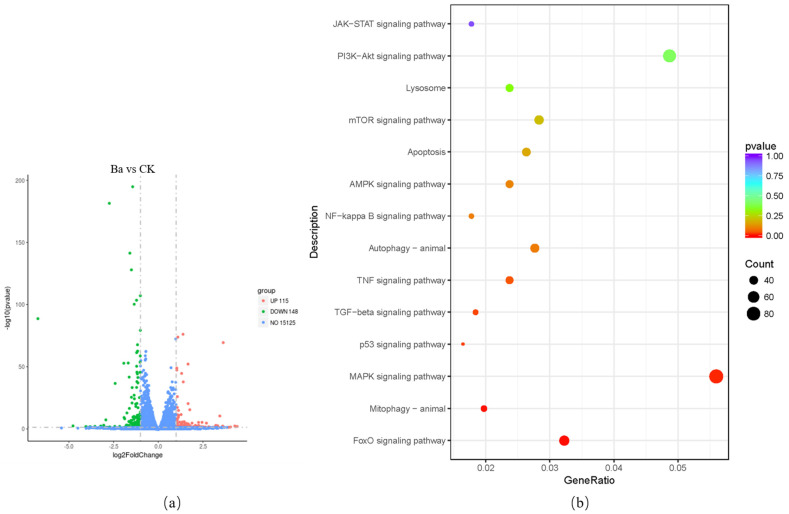

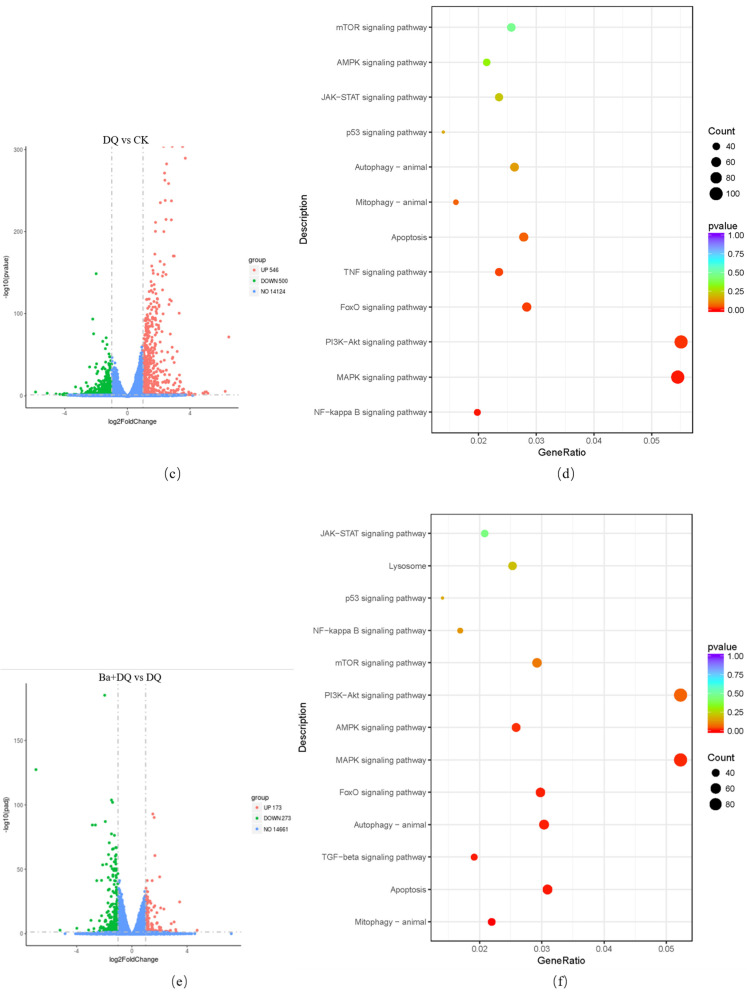

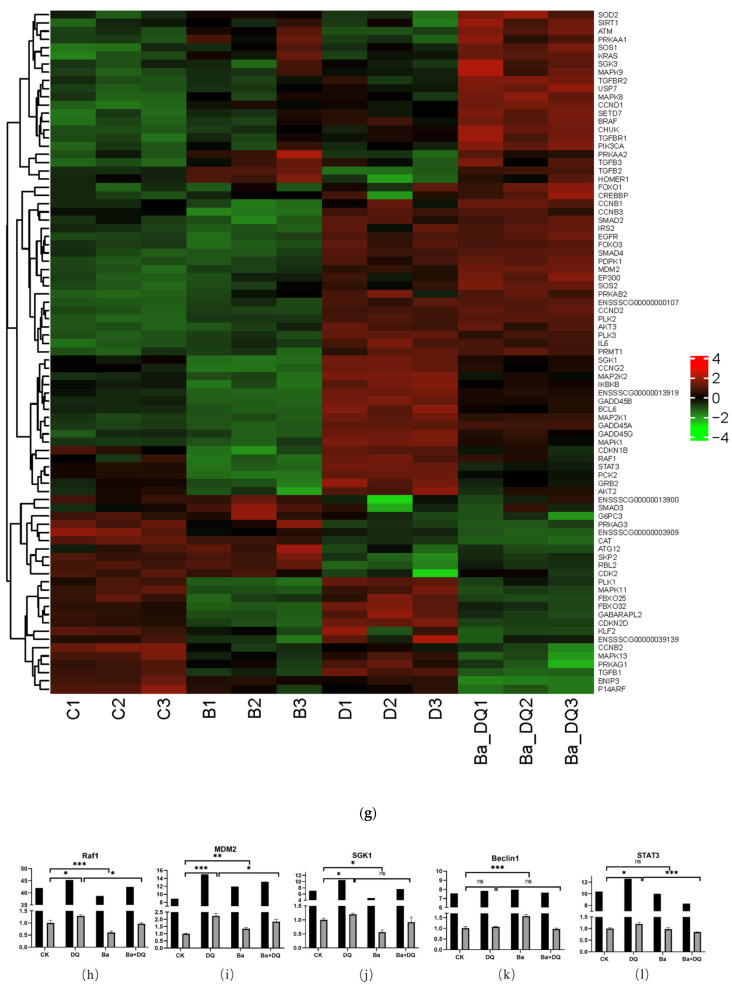
Upregulation and downregulation genes, and differential genes in the FOXO signaling pathway and validation of the DEGs data by RT-qPCR. (**a**–**f**) Volcano plots of the DEGs. The x-axis indicates the difference in expression level on a log2 scale (fold change), while the y-axis represents the *p*-value. Red represents the upregulation gene whereas green denotes downregulation genes. Bubbles of KEGG pathways for differential gene enrichment. The circle presents the gene number. The color of the circles indicates the *p*-value. (**g**) Heatmaps of all differential genes in the FOXO signaling pathway. (**h**–**l**) Validation of the RNA-Seq expression profiles of genes randomly selected from DEGG by RT-qPCR; *n* = 9 in each group. Black bars represent FPKM (Fragments Per Kilobase Million), while grey bars represent the fold change. FPKM normalized values as gene expression in RNA-seq. * *p* < 0.05, ** *p* < 0.01, *** *p* < 0.001, and ns = no significance (*p* > 0.05). Values are the mean ± SD.

The DEGs’ numerical analysis is depicted in Figure 5a,c,e. It was uncovered that compared to the CK group, *BaSC06* induced the upregulation of 115 and downregulation of 148 genes, while DQ induced the upregulation of 546 and downregulation of 500 genes. In addition, *BaSC06* upregulated 215 and downregulated 309 genes compared to the Ba+DQ group (fold change > 1 and *p* < 0.05). Then, a KEGG enrichment pathway analysis was performed and obtained the autophagy-related pathways, including MAPK, AMPK, PI3K-AKT, P53, FOXO, JAK-STAT, NF-kappa B, TNF, TGF-beta, and mTOR signaling pathways (Figure 5b,d,f). Among them, the FOXO signaling pathway was enriched with the most significant differential genes, which implies that this key pathway may regulate *BaSC06* or diquat-induced autophagy in IPEC-J2 cells. In addition, *BaSC06* was also found to have an effect on Akt in rats in our previous study [27], so we speculated that autophagy may be regulated by the Akt–FOXO signaling pathway.

Next, differential genes in the FOXO signaling pathway were examined, and 83 genes with significant differences were identified (Figure 5g). To verify the identified DEGs by qRT-PCR, we randomly selected 5 genes, SGK1, Beclin1, Raf1, MDM2, and STAT3, from the significant DEGs. The results revealed that the sequencing results were consistent with the RT-qPCR data, which confirmed the reliability of the sequencing data (Figure 5h–l).

### 3.6. BaSC06 Can Regulate the Transcriptional Activity of FOXO3 Transcription Factor in IPEC-J2 Cells

**Figure 6:**
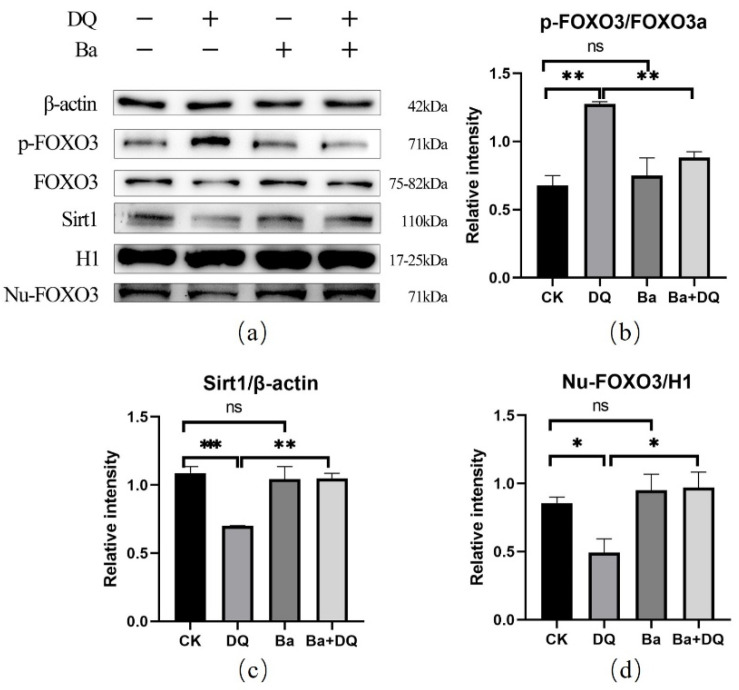
Effects of *BaSC06* on FOXO3 transcription factor in IPEC-J2 cells. (**a**–**d**) The expression of phosphate-FOXO3, FOXO3, and Sirt1 protein in IPEC-J2 cells. Cell lysates were collected to detect the protein levels of p-FOXO3/FOXO3, Nu-FOXO3/H1, and SIRT1/β-actin, and data analyses were executed using ImageJ software. Data were analyzed using one-way ANOVA with a Tukey test; *n* = 3 in each group; * *p* < 0.05, ** *p* < 0.01, *** *p* < 0.001, and ns = no significance (*p* > 0.05).

It is well known that the subcellular localization and transcriptional activity of FOXO proteins are mainly regulated by posttranslational modifications, including phosphorylation, acetylation, and ubiquitination [31]. Phosphorylation of FOXO3 can transfer it from the nucleus to the cytoplasm, thereby inactivating FOXO3. Currently, the expression of the p-FOXO3 protein in the DQ group was significantly increased (*p* < 0.01), which was reversed by the pretreatment of *BaSC06* (*p* < 0.01) (Figure 6a,b). More importantly, the expression of Sirt1 is crucial due to its deacetylation function. Noticeably, *BaSC06* significantly elevate the reduced expression of SIRT1 induced by DQ (*p* < 0.05) (Figure 6a,c), which implied that *BaSC06* may decrease acetylation of FOXO3. The protein expression FOXO3 in the nucleus decreased significantly in the DQ group (*p* < 0.05), while pretreatment with *BaSC06* inhibited this change in IPEC-J2 cells (Figure 6a,d). In summary, these outcomes indicate that *BaSC06* can increase the FOXO3 expression in nuclear by inhibiting its phosphorylation and increasing its deacetylation, thereby increasing the transcriptional activity of the FOXO3. 

### 3.7. BaSC06 Mediated Autophagy by the AKT–FOXO Signaling Pathway Independent of mTOR

**Figure 7:**
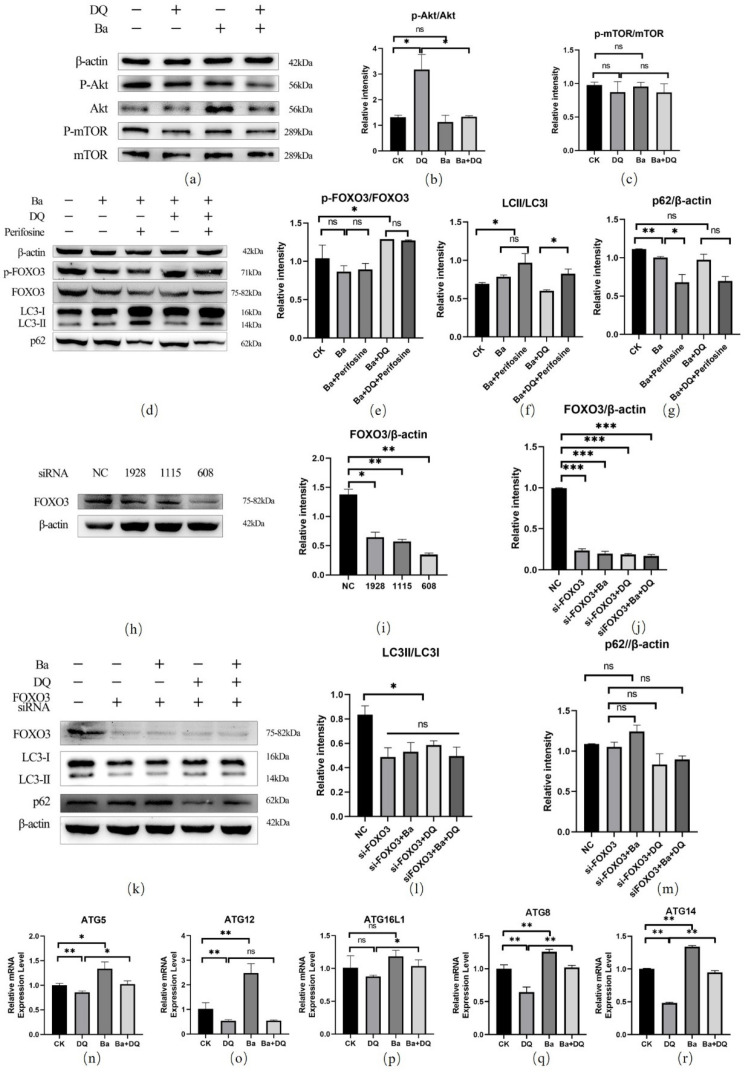
*BaSC06* mediated autophagy was triggered by the AKT–FOXO signaling pathway independent of mTOR, and the effect of *BaSC06* on FOXO downstream autophagy-related target genes. (**a**–**c**) The expression of p-AKT, AKT, p-mTOR, and mTOR protein in IPEC-J2 cells. Cell lysates were gathered to detect the ratio of *p*-AKT/AKT and p-mTOR/mTOR. (**d**–**g**) We added 25 μM perifosine into the *BaSC06* pretreated groups and collected the cell lysates to detect the protein levels of p-FOXO3/ FOXO3, LC3II/LC3I, and p62/β-actin. (**h**,**i**) The efficacy of all siRNAs. (**j**–**m**) The expression of FOXO3//β-actin, LC3II/LC3I, and p62 /β-actin in siRNA treatment groups. All data analyses were implemented using ImageJ software. Data were analyzed using one-way ANOVA with a Tukey test; *n* = 3 in each group; * *p* < 0.05, ** *p* < 0.01, *** *p* < 0.001, and ns = no significance (*p* > 0.05). (**n**–**r**) The relative mRNA expression levels of ATG5, ATG12, ATG8, ATG14, and ATG16L1. Data were evaluated using one-way ANOVA with a Tukey test; *n* = 9 in each group; * *p* < 0.05, ** *p* < 0.01, and ns = no significance (*p* > 0.05).

The ratio of p-AKT/AKT exhibited significantly reduced in the *BaSC06* pretreatment groups compared to the DQ group (*p* < 0.05) (Figure 7a, b). However, no significant change for the ratio of p-mTOR/mTOR was observed in all groups (*p* > 0.05) (Figure 7a,c). The results showed that the addition of perifosine (the inhibitor of AKT phosphorylation) significantly reduced the phosphorylation of FOXO3a (*p* < 0.05) (Figure 7d,e), confirming that the transcriptional activity of FOXO3a was upregulated. Then we used siRNA to reduce FOXO3 expression, and further detected the expression of autophagy related proteins in IPEC-J2 cells. SscFOXO3a-608 had the best silence effect (34.53 ± 0.16%), the protein expression level of FOXO3a was significantly reduced, so we used it in subsequent experiments (Figure 7h,i). The FOXO3/β-actin and the LC3II/LC3I ratio decreased significantly in sscFOXO3a-608-added groups (*p* < 0.05) compared to the NC group, but no significant difference was observed among all sscFOXO3a-608-added groups (*p* > 0.05) (Figure 7j–l). Although the expression level of P62 showed the increasing tend when Ba was added, there was no significant difference compared with NC group (*p* > 0.05). These results demonstrated that the decrease of FOXO3 expression significantly inhibited autophagy in IPEC-J2 cells, and that *BaSC06* induced autophagy by the AKT-FOXO signaling pathway, not by the AKT/mTOR signaling pathway. 

We further explored the expression of downstream autophagy-related target genes of FOXO. Our results demonstrated that compared with the CK group, the mRNA expressions level of ATG5, ATG12, ATG8, as well as ATG14 of the DQ group were substantially decreased, whereas these genes expression level significantly up-regulated in *BaSC06* treatment group (*p* < 0.05) (Figure 7n,o,q,r). Although treatments of DQ and *BaSC06* alone insignificantly affected the mRNA expression level of ATG16L1 (*p* > 0.05), pretreatment with *BaSC06* still markedly increased the mRNA expression level of ATG16L1 when it was compared with the DQ group (*p* < 0.05) (Figure 7p). These results verified that *BaSC06* could activate the AKT-FOXO signaling pathway to induce autophagy. 

## 4. Discussion

Diquat, which is widely used as an herbicide in agriculture, can be absorbed by green plants that turn it into peroxide-free radicals. Toxicological studies have demonstrated that diquat can cause damage to the digestive system as well as to the lung, liver, and other organs, eventually leading to death [30] and can induce oxidative stress and injuries in intestinal epithelial cells [32]. Therefore, Diquat has been largely applied to induce oxidative stress in vivo, because it can significantly increase the serum MDA level as well as inhibit the activities of antioxidant enzymes (including SOD and GSH-Px) [33,34,35]. But different cells have different sensitivities to it. 80 μmol/L DQ was used in our previous study in IEC-6 cells [27]. Current study found that the IC_50_ for DQ in IPEC-J2 cells was 932.2 μmol/ml, which is consistent with the results by Xu C et al. (2018), who used 1 mmol/L diquat to successfully establish an oxidative stress model on IPEC cells [36]. 

Elevated ROS production and low antioxidant capacity has been identified to induce oxidative stress in cells. MDA is an important biomarker of lipid peroxidation. Importantly, GSH-Px, CAT, and T-SOD are the main antioxidant enzymes in cells [37]. SOD terminates lipid peroxidation and eliminates ROS; however, its activity might be defected during acute injuries, leading to DNA damage, aggregated lipid peroxidation, and cell dysfunction [38]. The results of the present study showed that DQ exposure significantly increased MDA level in IPEC-J2 cells, while *BaSC06* pretreatment dramatically decreased the levels of ROS and MDA by reversing the decreased activities of SOD and GSH-Px induced by DQ, suggesting the potential role of *BaSC06* on alleviating oxidative stress, which corresponded with previous reports [27,36,39,40]. 

Evidence suggests that apoptosis can be caused by excessive oxidative stress, while excessive ROS production results in cell injury and death. Mitochondria are the major intracellular source of ROS [41]. The Bcl-2 protein family consists of Bcl-2, Bax, and Bak, among others. Bcl-2 is primarily located in the inner mitochondria membrane, the endoplasmic reticulum, and the perinuclear membrane and its function is regulated by its protein products, Bax and Bcl-xl [42]. What’s more, the Caspase family is a key protein of apoptosis [43]. Our results also revealed that DQ can induce cell apoptosis and damage in IPEC-J2 cells, which was indicated by the increased caspase-3 activity and the expression level of cleaved caspase-3 protein and the mRNA expression of caspase-8 gene, which is consistent with the results of our previous research on rats, *BaSC06* also decreased apoptosis of rats caused by diquat both in vivo and in vitro, which was verified by the reversal of the upregulated caspase-3 and Bax expression, and the decreased expression of Bcl2 [27]. Besides, DNA fragmentation has been found to occur during cell apoptosis. A study has reported that ROS reacts with cellular macromolecules through oxidation causing the cells to undergo an active process of cell death, which is set in motion by a high-conserved genetic program and culminated in DNA fragmentation and the formation of apoptotic bodies [44]. In this study, we verify that *BaSC06* can decrease apoptosis caused by diquat-induced oxidative stress via regulating the expression of apoptotic proteins and reducing the production of apoptotic bodies.

Autophagy is a catabolic pathway that is activated in response to different cellular stressors, such as damaged organelles, accumulation of misfolded or unfolded proteins, ER stress, accumulation of ROS, and DNA damage [45]. Several studies have reported that autophagy activation could alleviate oxidative stress. For instance, spermidine provides neuroprotection against oxidative stress and apoptosis by activating autophagy in aging male rats [46]. Tetrahedral framework nucleic acid inhibits chondrocyte apoptosis and oxidative stress through activation of autophagy [47]. In addition, quercetin helps the retina external barrier avoid oxidative stress injury by promotion of autophagy [48]. However, little information is available on the effect of probiotics on oxidative stress by regulating autophagy. Our previous study demonstrated that the autophagy induced by *BaSC06* was involved in decreasing oxidative stress in the rat [27]. Interestingly, we herein obtained similar results that *BaSC06* can upregulate the expression of LC3 and Beclin1, degrade p62, and increase LC3 puncta during diquat-induced oxidative stress in IPEC-J2 cells. Thus, it was suggested that the probiotic *BaSC06* contributes to alleviating oxidative stress by inducing autophagy in IPEC-J2 cells.

The results of KEGG enrichment pathway analysis revealed that DEGs and enriched major autophagy-related pathways contained P53, FOXO, JAK-STAT, NF-kappa B, TNF, TGF-beta, MAPK, AMPK, PI3K-AKT, and mTOR signaling pathways. In the Ba vs. CK, DQ vs. CK, and Ba+DQ vs. CK comparisons, FOXO was the primary signaling pathway that enriched the most significant DEGs regulating the autophagy process. The main DEGs in the FOXO signaling pathway are depicted in the heatmap included FOXO3, among others. Transcriptional factor FOXO3 has been uncovered to be extensively involved in autophagy and apoptosis, regulating the cell cycle, and participating in antioxidant stress response [49]. Moreover, FOXO3 activates autophagy by upregulating autophagy regulatory genes, including LC3, ATG12, γ-GABA receptor-associated protein 1 gene, yeast ATG8, and BNIP3 [50,51]. The acetylation, phosphorylation, and other post-transcriptional modification sites in FOXO significantly affect its DNA-binding activity, as well as its subcellular localization. The acetylation/deacetylation of FOXO regulates its transcriptional activity. Oxidative stress increase FOXO acetylation, and then acetylated-FOXO (Ac-FOXO) accumulates in the nucleus and binds to nucleosomes to block its transcriptional activity [52,53]. Furthermore, SIRT1 indirectly regulates autophagy by deacetylation of FOXO3, leading to increased expression of autophagy-related genes, including Bnip3, which are critical for autophagy induction [54]. The signal crossovers of SIRT1 and ROS can cause the decrease of autophagy and reduce the occurrence of the inflammatory response [55]. SIRT1 might deacetylate FOXO3 for oxidative stress response, thus improving the anti-stress ability of cells [56]. Overwhelming evidence suggests that SIRT1 deacetylates the FOXO factors, including FOXO1, FOXO3a, and FOXO4, and subsequently stimulate the expression of antioxidants, such as CAT, MnSOD, and Trx. Besides, an automatic feedback loop also potentiates SIRT1 expression [56,57,58,59,60]. In this work, *BaSC06* pretreatment significantly slowed the decline of SIRT1 induced by DQ, implying the increased deacetylation of FOXO3. 

Phosphorylation/dephosphorylation of FOXO determines its subcellular localization. Phosphorylation of FOXO is mainly affected by protein kinase B (PKB or AKT). Studies have shown that p-AKT can phosphorylate FOXO3 at Thr32 /Ser315 /Ser253, thus preventing FOXO3 from entering the nucleus and inhibiting autophagy gene transcription, thus down-regulating autophagy level [61]. Hence, AKT-FOXO3 primarily exerts a negative regulatory role on autophagy regulation. Reduced nucleation of FOXO3a leads to decreased expression of reactive oxygen scavenging enzymes (superoxide dismutase and catalase), which results in increased intracellular ROS. However, some studies have suggested that PI3K-AKT-FOXO3 can promote the level of autophagy flow. The underlying mechanism is that FOXO3, activated by phosphorylation, promotes the synthesis of glutamine synthase, and also prevents mTOR translocation to the lysosomal membrane in a glutamine synthase-dependent manner, causing mTOR inhibition, thus promoting autophagy [62]. Our results revealed that in the DQ group, the significant increased expression of p-AKT, p-FOXO3 and the significant decreased nuclear FOXO expression were observed, although no significant changes were found in the expression of mTOR. Nevertheless, pretreatment with *BaSC06* reduced the expression of p-FOXO3 and restored it to the same level as in the CK group. We therefore hypothesized that *BaSC06* could increase the expression of FOXO3 by inhibition of p-AKT, thereby inducing the autophagy of IPEC-J2 cells. To further verify this result, we treated IPEC-J2 cells with perifosine, an AKT inhibitor. Perifosine can significantly reduce the phosphorylation of AKT and then reduce the extracellular signal-regulated kinase (ERK) 1/2, inducing cell cycle stagnation in G1 and G2 [63]. Prolong treatment of breast cancer cells with AKT inhibitors (which inhibit AKT phosphorylation) induces dephosphorylation of FOXO3a, nuclear translocation, and destruction of its binding to SIRT6, resulting in FOXO3a acetylation and BRD4 recognition [64]. Our results showed that *BaSC06* and the inhibitor perifosine exhibited almost the same effect. This suggests that both *BaSC06* and the inhibitor perifosine might significantly reduce p-FOXO3 expression, increase LC3 expression, and promote the degradation of p62. In addition, our results showed that *BaSC06* could not upregulate the ratio of LC3II/LC3I after FOXO3 knockdown, suggesting that FOXO3 is necessary for *BaSC06* to induce autophagy in IPEC-J2 cells. Overall, these outcomes indicate that *BaSC06* can promote autophagy and antioxidant enzyme production in IPEC-J2 cells by inhibiting the AKT-FOXO signaling pathway, thus alleviating oxidative stress.

The extension of autophagic vesicles leads to the formation of autophagosomes, usually bilayer organelles. During the process of autophagy, there are two ubiquitination processes that occur in the ATG5-ATG12 and LC3 systems, which are essential for the extension of autophagic vesicles and the maturation of autophagosomes [65]. In particular, ATG5 is a key protein involved in phagocytic membrane elongation in autophagic vesicular, which forms a constituent complex with ATG12. In this process, ATG7 activates ATG12 as an E1-like ubiquitin activase. Then, ATG12 is delivered to the E2-like ubiquitin transferase ATG10. Eventually, ATG12 binds to ATG5 for forming a complex [66]. Afterward, the ATG12-ATG5 complex and ATG16L further form the ATG12-ATG5-ATG16L complex, which is found to be located on the outer membrane of the autophagosome. Of note, the ATG5-ATG12-ATG16L complex exhibits E3 ligase-like activity, essentially by activating ATG3 enzyme activity, promoting LC3 (namely ATG8) transfer from ATG3 to the bottom phosphatidylethanolamine (PE) [65]. The ATG5-ATG12-ATG16L complex is disintegrated from the membrane, once the autophagosome formed. Our results suggest that *BaSC06* may induce autophagy in IPEC-J2 cells by promoting the formation of the ATG12-ATG5 complex, while the increase of ATG12 may be activated by FOXO deacetylation. These outcomes indicated that *BaSC06* can promote the expression of autophagy-related genes by increasing FOXO activity.

## 5. Conclusions

**Figure 8:**
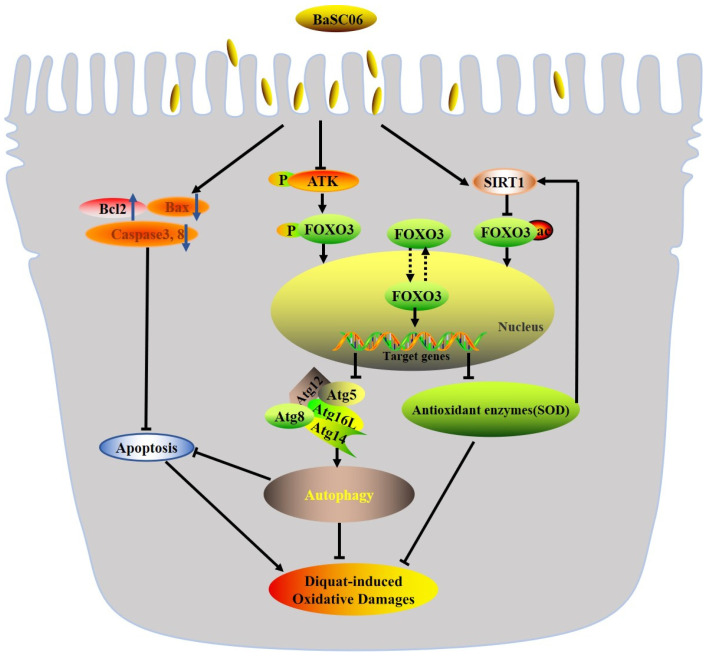
Proposed model of the protective effect of *BaSC06* in DQ-induced oxidative stress and apoptosis. (1) *BaSC06* inhibited the AKT-FOXO signaling pathway by inhibiting the expression of p-AKT, p-FOXO and increasing the expression of SIRT1, thereby increasing the gene expression of the ATG5-ATG12 complex to induce autophagy. (2) *BaSC06* attenuate apoptosis by modulating the activities of antioxidant enzymes, the expression of the apoptotic proteins Bcl2, Bax, Caspase 3 and Caspase 8 to alleviate oxidative stress.

Collectively, the possible molecular mechanisms underlying *BaSC06*-induced autophagy to attenuate oxidative stress are summarized as follows: *BaSC06* inhibited the AKT–FOXO signaling pathway by decreasing the expression of p-AKT and p-FOXO and increasing the expression of SIRT1, thereby increasing the transcriptional activity of FOXO3 and gene expression of the ATG5–ATG12 complex, which induce autophagy to alleviate oxidative stress in IPEC-J2 cells. Besides, *BaSC06* attenuates apoptosis by modulating the activities of antioxidant enzymes and the expression of the apoptotic proteins Bcl2, Bax, Caspase 3 and Caspase 8. All the above findings provide a valuable theoretical basis for the application of probiotics in preventing and treating diseases caused by oxidative stress and improving human and animal health (Figure 8).

## Figures and Tables

**Table 1:** List of siRNA.

Gene Name.	Access No.	Sequences	
		Sense(5′-3′)	Antisense(5′-3′)
sscFOXO3a-608	NM_001135959.1	CCGGCUGGAAGAACUCUAUTT	AUAGAGUUCUUCCAGCCGGTT
sscFOXO3a-1115	NM_001135959.1	CCGGAACCAUGAAUCUCAATT	UUGAGAUUCAUGGUUCCGGTT
sscFOXO3a-1928	NM_001135959.1	CCCUCAUCUCCACACAGAATT	UUCUGUGUGGAGAUGAGGGTT
Negative Control	NM_001135959.1	UUCUCCGAACGUGUCACGUTT	ACGUGACACGUUCGGAGAATT

**Table 2:** List of qPCR primers.

Gene	Access No.	Primers Sequence	Length
*GAPDH*	NM_001206359.1	F: CGGAGTGAACGGATTTGGC	248
		R: CACCCCATTTGATGTTGGCG	
*ATG5*	NM_001037152.2	F: AGTCAACCCTCCAATACCCAG	299
		R: TGTGGCCCTCTCTAGGTTTCT	
*ATG12*	NM_001190282.1	F: AGGTTGGATACCCGCCTACT	111
		R: ACTTGGTTGGAGCAATCT	
*ATG16L1*	NM_001190272.1	F: CTGCCAGTCGAACAGGATGA	166
		R: AGCGCCTCCCAAAGATATTAGT	
*ATG8*	NM_001190288.1	F: CCACCTTCCCACTCAGCTTT	187
		R: GTGTATCCTACCTTCCCCGC	
*ATG14*	XM_001924990.5	F: GCTTACACTGGACACCGCTA	125
		R: CCCCACGGCTTAACCTCTTT	
*Caspase-8*	NM_001031779.2	F: CCAGGATTTGCCTCCGGTTA	108
		R: GCCAGGTCATCACTGTCCAA	

## Data Availability

All data that support the findings of this study are available in the submitted article except the RNA-seq, and the RNA-seq data are not publicly available due to privacy. The data are available from the corresponding author upon reasonable request.
